# Antiseptic Collodion

**Published:** 1887-06

**Authors:** 


### Antiseptic Collodion.

The *Journal de Medecine* of December 26 contains an account of a new kind of "collodion," which is antiseptic and promotes cicatrization. It does not cause inflammation, and may be substituted for collodion made from gun cotton, in the treatment of wounds and bruises. Like traumaticine, it is efficacious in relieving neuralgic pains, and acute or chronic rheumatism. The affected parts should be sponged with it every twenty-four hours, and in serious cases every six hours. If strips of linen or silk be soaked in this collodion, an excellent sticking plaster is obtained, which quite equals English court-plaster. The following is the formula; Mastic in globules, 3 grammes; balsam of Peru, 1 gramme; narcotine, 1 gramme. Each ingredient should be ground separately, and 5 grammes of chloroform added thereto.

